# Forensically relevant anatomical brain regions cannot be sub-differentiated by RNA expression analysis

**DOI:** 10.1007/s12024-024-00787-7

**Published:** 2024-01-31

**Authors:** Jan Euteneuer, Lucas Moitinho-Silva, Cornelius Courts

**Affiliations:** 1 Institute of Forensic Medicine, University Hospital of Schleswig-Holstein, Arnold-Heller-Strasse 12, 24105 Kiel, Germany; 2 Institute of Clinical Molecular Biology, University of Kiel, Kiel, Germany; 3 Institute of Legal Medicine, University Hospital of Köln, Melatengürtel 60/62, 50823 Cologne, Germany

**Keywords:** Forensic genetics, Organ tissue identification, RNA analysis, RNA sequencing, Head trauma, Brain regions

## Abstract

The contextualization of biological traces generated by severe head injuries can be beneficial for criminal investigations. Here we aimed to identify and validate mRNA candidates for a robust sub-differentiation of forensically and traumatologically relevant brain regions. To this purpose, massively parallel sequencing of whole transcriptomes in sample material taken from four different areas of the cerebral cortex (frontal, temporal, parietal, occipital lobe) was performed, followed by bioinformatical data analysis, classification, and biostatistical candidate selection. Candidates were evaluated by Multiplex-RT-PCR and capillary electrophoresis. Only a weak relative upregulation and solely for candidates expressed in the parietal lobe was observed. Two candidates with upregulation in the cerebellar region (PVALB and CDR2L) were chosen for further investigation; however, PVALB could not reliably and repeatedly be detected in any lobe whereas CDR2L was detectable in all lobes. Consequently, we suggest that differences in mRNA expression between four regions of the cerebral cortex are too small and less pronounced to be useful for and applicable in forensic RNA analysis. We conclude that sub-differentiation of these brain regions via RNA expression analysis is generally not feasible within a forensic scope.

**Supplementary Information:**

The online version contains supplementary material available at 10.1007/s12024-024-00787-7.

## Introduction

The identification of forensically relevant biological material by RNA analysis can provide valuable contextual information on trace patterns to support investigations in cases where DNA analysis alone is not sufficient [1]. Currently, the detection of body fluids and organ tissues, including brain, even in challenging forensic sample material is a valid and routinely used approach for trace contextualization [2, 3].

It has been shown that RNA can be accessible to forensic molecular analysis even when isolated from traces of backspatter collected from inside parts of firearms after shooting [4, 5]. “Backspatter” is the term used for biological traces generated by the propelling of droplets and spatters of blood and tissue fragments back from the entrance wound in a body caused by a projectile into the direction of the firearm and the shooter [6]. The analysis of nucleic acids isolated from backspatter in “molecular ballistic” investigations [7] was successfully used to identify victims shot by a specific firearm [8]. Also, it was shown that a suspected shot to the head can be inferred by detecting brain specific RNA in traces of backspatter [9].

In this context, in cases of severe head traumata caused by high-energy firearm projectiles or massive blunt impacts, for example, in situations when multiple offenders caused injuries to the same victim handling different weapons, it can be beneficial for criminal investigations to determine which specific area of the head had been hit (and by which object), by proxy of employing the RNA analysis of trace material to detect which part of the brain had been injured. Such an approach could then be implemented into established forensic analysis routine workflows and could include the identification of messenger-RNA (mRNA) as well as small non-coding RNA, e.g., micro-RNA (miRNA).

In this study, we performed whole transcriptome sequencing on a training set of samples of autoptic brain tissues to identify mRNA markers specific for four different forensically and traumatologically relevant areas of the cerebral cortex (frontal, temporal, parietal, and occipital lobe). The resulting differentially expressed RNA candidates were then evaluated in terms of sensitivity, specificity (using available published datasets), and their technical suitability in applied forensic trace analysis. A further goal was to establish whether reliable analytical sub-differentiation of functionally different but anatomically very similar tissues by RNA sequencing under forensic requirements is feasible at all.

## Material and methods

### Sample collection

Samples from 5 males and 5 females (age range 26–68 years, median: 56.5) of about 0.5 cm^3^ in size were excised by expert forensic pathologists during medico-legal autopsies from both hemispheres in distinct, forensically relevant positions: (1) frontal lobe (most anterior part of Brodmann area 10), (2) parietal lobe (inferior part of the postcentral gyrus about 3 cm superior of the lateral sulcus), (3) temporal lobe (auditory cortex), and (4) occipital lobe (occipital pole at the most posterior part of Brodmann area 17). This set of samples is referred to as “first set.” A separate second set of three samples (1 female, 2 male) was later collected as described above. Individuals with prolonged post mortem interval, head or brain trauma, signs of putrefaction, or known or visible neurodegenerative diseases were excluded from the study. All tissue samples were directly transferred to RNAlater™ Stabilization Solution (Thermo Fisher Scientific, USA) and stored at − 80 °C until use.

### RNA extraction and preparation for RNA sequencing

To avoid contamination and RNA degradation, all steps were performed wearing protective gear including sterile surgical gowns (Lohmann & Rauscher, Germany), standard earloop facemasks (Moss GmbH, Germany), nurses caps (Mölnlycke Health Care, Sweden), and Micro-Touch^®^ nitrile examination gloves (Ansell, Belgium) and all equipment and working spaces were cleaned before and repeatedly during the workflow with 10% bleach (DanKlorix, Colgate-Palmolive, Germany), 70% ethanol (Th. Geyer GmbH & Co. KG, Germany), and additional RNaseZap™ Solution (Thermo Fisher Scientific).

Total RNA was extracted from about 50 mg of brain tissue from each sample using the mirVana™ miRNA Isolation-Kit (Thermo Fisher Scientific), according to the manufacturer’s protocol, except for the second set of samples used for the PVALB and CDR2L assays, where the “Homogenate Additive” was omitted after consulting with the manufacturer and modifying the RNA extraction workflow, based on the information that the “Homogenate Additive” only increases the yield in small RNAs, but not mRNA. Subsequent DNase digestion was performed using the TURBO™ DNA kit (Thermo Fisher Scientific) according to the manufacturer’s protocol for “rigorous DNase treatment.” RINs (RNA integrity numbers) were obtained employing the RNA 6000 Nano Assay protocol according to the manufacturer’s recommendations on a 2100 Bioanalyzer (Agilent Technologies, USA).

### RNA library preparation, sequencing, and data processing

mRNA sequencing (25 M reads) was performed at the Institute of Clinical Molecular Biology (IKMB) in Kiel, Germany, on a NovaSeq S1 (Illumina, USA) after TruSeq stranded mRNA (Illumina) library construction. Raw sequences were processed using the nf-core/smrnaseq v3.4 [10], which includes adapter quality trimming with Trim Galore (https://github.com/FelixKrueger/TrimGalore). Ribosomal RNA sequences were removed applying SortMeRNA [11]; alignment to the reference genome (GRCh38, Ensemble 104) was done with STAR [12] and quantification by using Salmon [13]. Differentially expressed genes were detected with the R package DESeq2 version 1.32.0 [14]. Likelihood-ratio tests were performed with linear models with negative binomial distribution. *p* value adjustment for multiple testing was completed using the Benjamini and Hochberg method. Effect sizes were shrank using the R package apeglm [15].

### Candidate selection and primer design

For each brain location, the obtained genes were compared to every other location. Filtering was done for a set of possible robust and area specific mRNA candidates by the following selection criteria: a. increased expression, i.e., upregulation compared to all other lobes (and sites); b. significance, i.e., adjusted *p* value < 0.01; c. robustness, i.e., BaseMean > 50; and d. log2FoldChange > 1.

After filtering, remaining mRNA candidates were cross-checked for known expression patterns with The Human Protein Atlas (https://www.proteinatlas.org/) and BioGPS (http://biogps.org/). Primer design for candidate genes was done with Primer-BLAST (https://www.ncbi.nlm.nih.gov/tools/primer-blast/), with amplicon size and melting temperature adapted fitting to the in-house used mRNA assay (primer sequence shown in Supplementary File).

### Reverse transcription, amplification, and capillary electrophoresis

cDNA was synthesized employing the High-Capacity cDNA Reverse Transcription Kit (Thermo Fisher Scientific) according to the manufacturer’s protocol with a total RNA input of 20 ng. PCR assays with optimized primer pairs at 0.1 µM each and additional 1 µM (PVALB) input concentration in addition to the reference marker 18s-rRNA (0.008 µM) were run using the QIAGEN Multiplex PCR Kit (Qiagen, Germany) with 1 ng cDNA input and the following PCR settings: initial HotStarTaq DNA polymerase activation for 15 min at 95 °C, followed by 33 cycles of a. 20 s at 94 °C, b. 30 s at 64 °C, and c. 40 s at 72 °C. Final elongation was performed for 45 min at 60 °C. Post-PCR purification was done by gel filtration employing the illustra™ Sephadex™ G-50 Fine DNA Grade powder (Cytiva, USA). Amplicons were separated and detected on a 3500 Genetic Analyzer (Thermo Fisher Scientific) employing LIZ500 size standard (Thermo Fisher Scientific) and according to an in-house validated and accredited RNA analysis protocol. Data analysis was done using the GeneMapper ID-X software (Thermo Fisher Scientific) version 1.6.

## Results and discussion

Brain samples from four distinct cerebral cortex regions of both hemispheres were collected during medico-legal autopsies, aiming for an even distribution of male and female samples. The final set comprised 80 samples with a PMI from 4 up to approximately 9 days. Quality control of the extracted total RNA resulted in mean RIN values between 2.4 and 4.9 (Table 1). No correlation between RIN, age, and PMI was observed.

**Table 1:** Overview of age, sex, PMI, and measured RIN from used autopsy cases

**Set/use**	**Brain index no.**	**Age**	**PMI [d]**	**Sex**	**Mean RIN (SD)**
**1st/RNA sequencing**	2	63	7	M	2.7 (0.25)
	4	26	6	F	4.3 (0.58)
	7	35	8	M	4.0 (0.51)
	8	57	5	F	4.9 (0.57)
	10	68	7	M	2.4 (0.31)
	11	65	9	F	4.1 (0.92)
	13	56	5	F	4.5 (1.12)
	14	60	5	M	3.9 (0.83)
	16	47	6	F	4.9 (0.74)
	17	49	4	M	3.6 (1.04)
**2nd/PVALB/CDR2L**	6	57	8	M	n.p.
	9	80	6	F	n.p.
	15	52	2	M	n.p.

Collected data from two sets of autopsy cases and respective brain samples. RIN values are means of values obtained from the samples of the distinct areas

*PMI* post mortal interval, *[d]* days, *M* male, *F* female, *RIN* RNA integrity number, *SD* standard deviation, *n.p.* not performed

After whole transcriptome sequencing, a comprehensive bioinformatic data analysis was performed and statistical classification algorithms were applied to identify robust mRNA candidates with upregulated expression that is significantly differential between the different brain areas. Briefly, using the above described data processing algorithms and tools, the transcriptome expression was tested for significant differential expression between any level of variable, i.e., lobe and hemisphere, and for each location against all others separately. From 1,030,286 differentially regulated RNAs detected in the dataset, filtering according to the above-mentioned selection criteria identified 29 candidates, which all exhibited low log2FoldChange values between 1 and 1.9, indicating only a weak upregulation, however. Notably, all 29 candidates were identified in samples from the parietal lobe, leading to the conclusion that a sub-differentiation of all four regions based on RNA expression was not feasible.

Therefore, we assessed the potential of our data to differentiate parietal lobe from non-parietal lobe regions which would still be of forensic interest given that the parietal region corresponds to the main hit zone of the skull for homicidal gunshots in Germany [16]. Hence, all 29 selected mRNA candidates were checked via database surveys (BioGPS, The Human Protein Atlas) for known expression patterns in other tissues using those available sequencing data sets from non-forensic brain samples, which showed all candidates to be either evenly expressed over every cerebral cortex region or to be upregulated in more than one region. Still, two candidates with distinct levels of upregulation in the cerebellar region according to the available datasets, parvalbumin (PVALB) and cerebellar degeneration related protein 2 like (CDR2L), were chosen for further investigation, as their corresponding region of expression (cerebellum) is anatomically associated with the back of the head. Brain samples (Table 1) of the second set were then prepared for analysis, and RNA expression of PVALB and CDR2L together with a commonly used positive control marker (18s-rRNA) was measured utilizing a set of newly designed primers (see Supplementary Material).

**Fig. 1:**
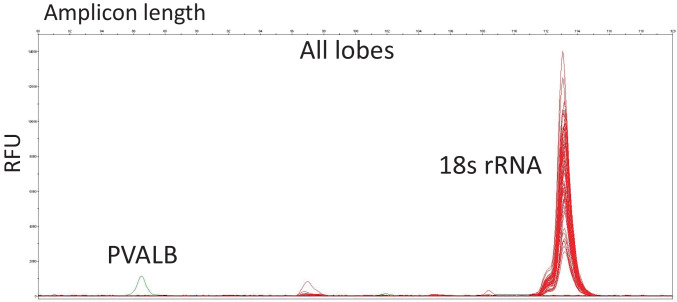
Expression of PVALB. Combined depiction of electropherograms of the expression of PVALB and 18s rRNA in all brain regions (frontal lobe, occipital lobe, parietal lobe, temporal lobe) in a set of three brain samples. X-axis depicts amplicon length (80–120 bp); Y-axis depicts RFU (relative fluorescent units, between 0 and 15,000)

**Fig. 2:**
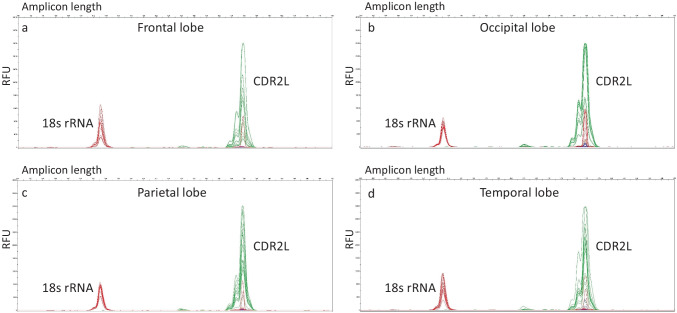
Expression of CDR2L. Electropherogram depictions of the expression of CDR2L and 18s rRNA in investigated brain regions, each combined for a set of three brain sample. X-axes depict amplicon length (100–150 bp); Y-axes depict RFU (relative fluorescent units, between 0 and 40,000). **a** Expression of CDR2L and 18s rRNA in frontal lobe, **b** expression of CDR2L and 18s rRNA in occipital lobe, **c** expression of CDR2L and 18s rRNA in parietal lobe, **d** expression of CDR2L and 18s rRNA in temporal lobe

Analysis of the fresh set of brain samples showed that PVALB did not exhibit reliable and stable expression in any of the investigated brain regions (Fig. 1), whereas CDR2L expression was detectable in all regions (Fig. 2). Replicate analyses after adjustments of primer concentrations and PCR parameters produced the same results (data not shown).

While new mRNA candidates are still being discovered and added to existing multiplex assays for the identification of forensically relevant tissues and body fluids, such as rectal mucosa [17, 18], an attempt to differentiate tissues or organ sub-regions as similar as the four cortical areas focused upon in this study has not been taken so far, to the best of our knowledge. Apparently though, the differences in biology and gene expression between these regions are too exiguous to meet the requirements to be set for forensic RNA analysis, i.e., robust and specific (i.e., strongly differential) expression that is so abundant that it can compensate for a decrease in intensity due to aging, degradation, and other factors typically challenging forensic type samples and workflows.

Further research could assess the potential of other molecular biological techniques such as forensic epigenetics, e.g., DNA methylation analysis, to differentiate samples from different cortical sub-regions. DNA methylation analysis is a subject of active forensic research and is routinely used in forensic age estimation (summarized, e.g., in [19]). Also, in this context, brain tissue had already been included in a study of tissue specific methylation patterns [20], while non-forensic studies of epigenetic variation of the human brain show potential for further investigations [21].

Lastly, forensic proteomics may be another alternative to identify and sub-differentiate biologically similar tissues; two current articles review promising data available so far [22, 23].

## Conclusion

From the data and samples included in this study, no mRNA candidate marker to sub-differentiate forensically relevant but biologically similar cortical regions could be identified that fulfilled the criteria for markers to be used in forensic RNA analysis. Whether increasing the number of investigated brain regions and/or sample size would produce better results and/or suggest different mRNA candidates for the task remains unknown. Based on currently available data, however, we conclude that sub-differentiation of the four brain regions included in this study via RNA expression analysis is not feasible within a forensic scope.

## Key points

1. Whole transcriptome sequencing of samples from four forensically relevant brain regions.
2. mRNA candidate selection based on bioinformatic processing.
3. No differential gene expression between brain regions.
4. Sub-differentiation of brain regions via RNA expression analysis is out of forensic scope.

## Supplementary Information

Below is the link to the electronic supplementary material.

Supplementary file1 (XLSX 11 KB)

## Data Availability

Raw and additional data are available upon request.

## Declarations

### Previous or partial publication of contents

Preliminary results from this study were presented at the 29th Congress of the International Society for Forensic Genetics, Washington, DC, USA, August 29th to September 2nd, 2022 (P209). Concomitantly, a non-peer-reviewed “extended abstract” titled “Towards localizing head shots – Forensic sub-differentiation of anatomical brain regions by differential RNA expression” was published in the Forensic Science International: Genetics Supplement Series (10.1016/j.fsigss.2022.10.027), the corresponding publication venue for the proceedings of scientific symposia.
